# Hormone Replacement Therapy in Cancer Survivors – Review of the Literature

**DOI:** 10.1007/s12253-018-00569-x

**Published:** 2019-01-08

**Authors:** Tamás Deli, Mónika Orosz, Attila Jakab

**Affiliations:** Department of Obstetrics and Gynecology, Faculty of Medicine, University of Debrecen, Debrecen, Hungary

**Keywords:** Hormone replacement therapy, HRT, Menopausal hormone therapy, MHT, Estrogen replacement, Estrogen-progestin replacement, Oncologic patient, Cancer survivor, Gynecologic cancer, Non-gynecologic cancer, Breast cancer, Recurrence risk

## Abstract

Rapid advance in oncology leads to increasing survival of oncologic patients. More and more of them live long enough to reach either the natural age of menopause or, as a side effect of their oncotherapy, experience the cessation of gonadal function, leading to premature ovarian insufficiency, with disturbing vasomotor symtoms and long-term negative cardiovascular and skeletal effects. Thus, an ever increasing number of cancer survivors search endocrinologic help in the form of hormone replacement therapy (HRT). The misinterpretation of the WHI (Women's Health Initiative) Study has lead to an irrational fear of female hormone replacement, both by the general population and medical professionals. It has seemed the logical and safe conclusion to many physicians to avoid HRT, supposing that this attitude definitely causes no harm, whereas the decision of prescribing estrogen alone or with progestins might bear oncologic and thromboembolic risks and may even lead to litigation in case of a potentially related complication. However, it was known even before the WHI results that premature menopause and hypogonadism decreases the life expectancy of women by years through its skeletal and cardiovascular effects, and this negative effect correlates with the length of the hypoestrogenaemic period. Therefore, the denial of HRT also needs to be supported by evidence and should be weighed againts the risks of HRT. Yet, the oncologic risk of HRT is extremely difficult to assess. In this work we review the latest evidence from in vitro experiments to clinical studies, regarding HRT in survivors of gynecologic and non-gynecologic cancers. Based on our literature research, we group tumours regarding the oncologic risk of properly chosen female hormone replacement therapy in cancer survivors as follows: ’HRT is advanageous’ (e.g. endometrial cancer type I, cervical adenocarcinoma, haematologic malignancies, local cutaneous malignant melanoma, colorectal cancer, hepatocellular cancer); ’HRT is neutral’ (e.g. BRCA 1/2 mutation carriers without cancer, endometrial cancer type II, uterinal carcinosarcoma and adenosarcoma, certain types of ovarian cancer, cervical, vaginal and vulvar squamous cell carcinoma, prolactinoma, kidney cancer, pancreatic cancer, thyroid cancer); ’HRT is relatively contraindicated’ for various reasons (e.g. leiomyosarcoma, certain types of ovarian tumours, brain tumours, advanced metastatic malignant melanoma, lung cancer, gastric cancer, bladder cancer); ’HRT is diasadvantageous and thus contraindicated’ (e.g. breast cancer, endometrial stroma sarcoma, meningioma, glioma, hormone receptor positive gastric and bladder cancer).

## Introduction

Hormone replacement therapy (HRT; also known as menopausal hormone therapy, MHT) means substituting estrogen (or compounds exerting estrogenic effects) and progesterone (or compounds exerting progestagenic effects) after the cessation of cyclic ovarian hormone production. In the context of young oncologic patients with premature ovarian insufficiency (POI) the term HRT seems to be more appropriate then MHT. Oncologic risk of MHT requires consideration from two aspects: the potential of MHT to induce tumours in patients who have no oncologic history; and the potential to cause cancer recurrence and progression in cancer survivors. The former question is frequently asked by patients and needs to be explained to those who receive MHT. To date, a lot of data from large randomised controlled trials and relevant guidelines are available in context of the most common malignancies, such as breast or colorectal cancer. Most of these are reviewed in the clinical guidelines of menopausal hormone therapy, such as that of the International Menopause Society, IMS [1], the National Institute for Health and Care Excellence, NICE [2] or the American College of Obstetricians and Gynecologists, ACOG [3].

The situation is far more complex when it comes to cancer survivors. They can experience premature ovarian insufficiency as a consequence of cancer treatment (surgery, chemotherapy or radiotherapy), or as a result of an independent disease (see all the possible causes of POI, e.g. genetic or autoimmune diseases, or the consequence of other benign ovarian pathology), or simply may survive long enough to reach the age of physiological menopause around the age of 50. HRT may be necessary because of menopausal symptoms, but young asymptomatic patients should also receive hormone replacement if not contraindicated. It is well known that refusal of MHT decreases not only the quality of life, but also the life expectancy of young menopausal patients by several years. In a Dutch study, this was found to be 2 years lost over a 17-year follow-up period, mainly due to cardiovascular and osteoporotic morbidity [4]. Thus, not only inadequate initiation of MHT, but also its unsubstantiated denial, ’just to be on the safe side’, harms the patient.

Professional decision-making, however, is hindered by several factors. The major problem is the nearly infinite number of possibilities that biologically characterize the two potentially counteracting participants: the malignant disease and hormone therapy. Our decision depends on

- the general *oncologic characteristics of the malignant disease* and its former or current therapy (the organ affected, histologic type, molecular oncologic characteristics, grade and stage of tumour, therapy applied, stage of remission, time of survival since therapy);
- the *specifications of the planned hormone substitution therapy* (estrogenic and progestagenic compound type, dose, sequential or continuous fixed combination regimen, route of administration, duration of MHT);
- and the oncologically relevant *endocrine caracteristics of the tumour*, e.g. hormone receptor status (presence of hormone receptors, receptor subtypes, receptor splice variants, or estrogen-related receptors), former or current endocrine oncotherapy (aromatase inhibitors, selective estrogen receptor modulators, selective estrogen receptor degrader, GnRH analogues) or the effect of female hormones on the given tissue in general, or on the specific tumour type in particular. It is even possible, that in vivo HRT exerts its effect on tumour recurrence and progression (even contradicting in vitro results on isolated cells or tumour tissues) not by affecting the malignant cells themselves, but rather influencing for example the surrounding stromal tissue, the immune response of the body, or cells and structures participating in metastatization.

It is obvious from this list that although ’individualized decision-making’ is needed, data on which this should be based can not always be readily available. When trying to find relevant data, depending on the prevalence of the tumour type, papers from preclinical research, case reports, retrospective studies, randomized controlled trials and meta-analyses can be found. Their strength to predict the risks and benefits of MHT in the given clinical setting is varied, yet, these may be the only source of information for the clinician.

In this review we try to summarize the data regarding the advantages and disadvantages of HRT in the most common tumour types.

## Determinants of Estrogen Effect on Tissues

**Fig. 1:**
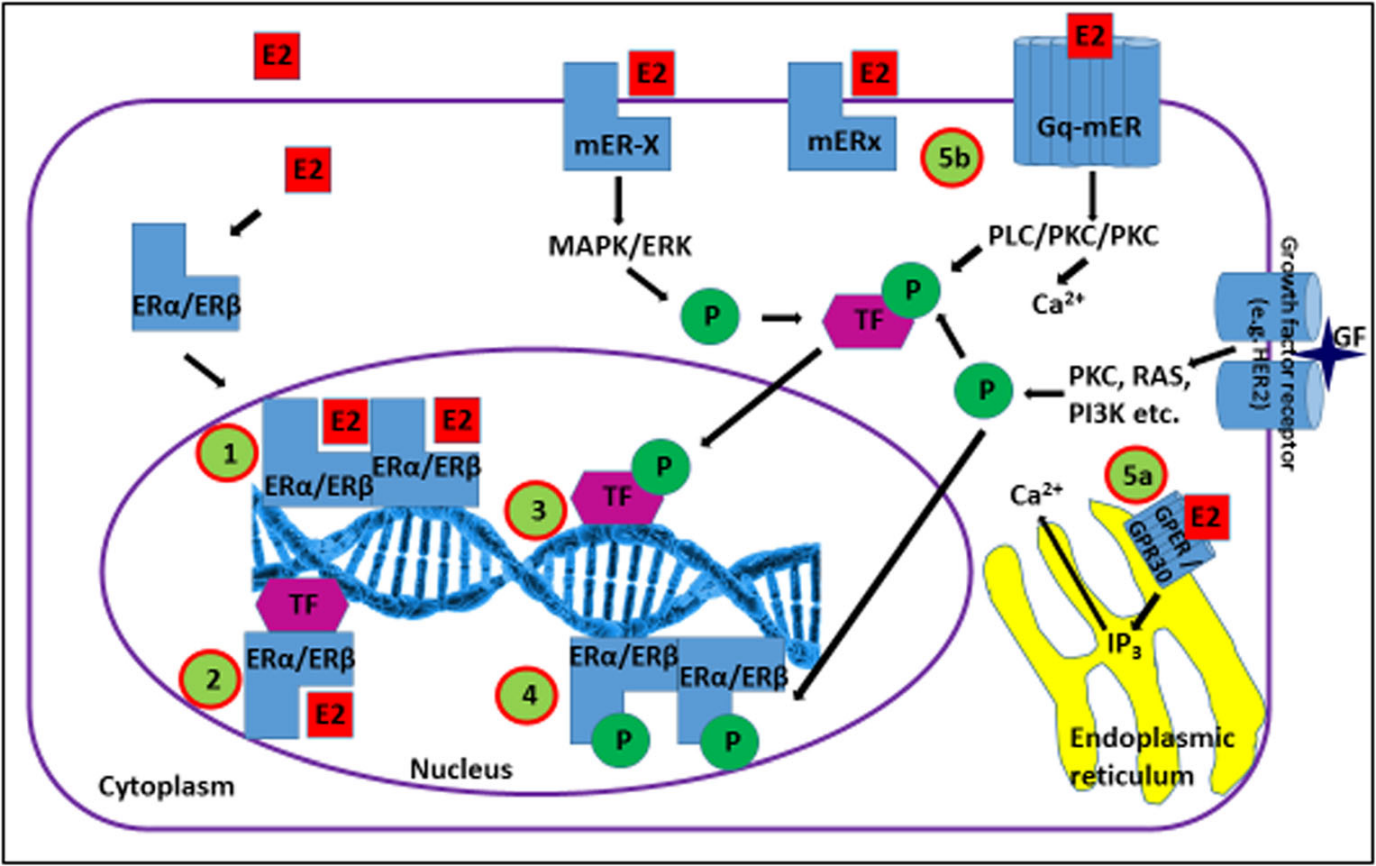
**Modes of action of estrogen receptors**. Estrogen receptors can exert their effects in several ways. **1.** Estrogen + nuclear ER, dimerization, direct DNA binding. **2.** Estrogen + nuclear ER + transcription factor, DNA binding. **3.** Phosphorylation of transcription factor through an estrogen dependent or independent pathway, DNA binding without ER. **4.** Phosphorylation of nuclear ER through an estrogen dependent or independent pathway, DNA binding without estrogen ligand. **5a.** Non-genomic effect of estrogen via GPER endoplasmic reticulum membrane bound ER, Ca^2+^ signalling activated. **5b.** Non-genomic effect of estrogen via cell surface membrane bound mERs, Ca^2+^ signalling and kinases activated. E2: estrogen, ER: estrogen receptor, mER (mER-X, mERx): membrane bound estrogen receptor (-X and x), Gq-mER: G-protein coupled membrane bound estrogen receptor, GPER/GPR30: G-protein coupled estrogen receptor 1 / G-protein coupled receptor 30, TF: transcription factor, GF: growth factor

The effect of estrogen on a tissue or a certain cellular function is not at all straightforward. Classic *nuclear estrogen receptors* (ERs), ERα and ERβ exert their effect after estrogen binding and dimerization via binding to estrogen response elements (ERE) in the promoter and regulatory regions of their target genes [Fig. 1]. This binding can be direct or can be mediated by *transcription factors* (TFs). Also, *ligand (estrogen) independent activation* and ERE-binding of ERs via phosphorilation triggered by for example growth factor receptors (e.g. HER2, IGF-1R), as well as *ER independent activation* and ERE-binding of TFs is known. This activation can be mediated by endoplasmic reticulum or surface *membrane bound classic or G-protein-coupled 7-transmembrane spanning (GPER or GPR30, xER types) receptors*, or estrogen independent protein kinase activation. [5, 6]

Estrogen sensitivity and ERE mediated activation of cells or genes can be defined in several ways. When gene upregulation following the addition of estrogen is measured, over 1000 estrogen sensitive genes can be identified, the exact number depending on cell and tissue type [7]. The other approach is identifying EREs in the promoter region of genes. ERE databases of the human genome are available online [8, 9], and include for example the following genes: *EBAG9*, c-*fos, OXT, F12, TFF1, LTF, CTSD, PFDN2, TGF-*α, *AGT, GREB1,* KIAA1243, NRIP1, MADH9, NME3, TPD52L, and ABCG2. [9].

To make the situation even more complex, *estrogen-related receptors* (ERRα, ERRβ, ERRγ), several estrogen-binding and non-estrogen-binding *splice variants (isoforms)* of ERα (e.g. ERα-46, ERα-36) and ERβ (ERβ1-5), and interaction of ERα and ERβ via *heterodimerization* have been known for several years [10, 11]. Taken all these into consideration, it is clear that the presence of estrogen and its receptor is not enough to predict the effect of estrogen in any cell type. Yet, the estrogen (and progesterone) receptor status has long been known to be an important prognostic factor and has been used to determine the mode of oncotherapy in certain cancer types, for example breast cancer. Estrogen receptor overexpression, however, has not only been found in the well-known estrogen dependent tumour types, such as breast, endometrial and ovarian cancer. Altered estrogen and / or progesterone receptor expression has been documented for example in thyroid cancer [12–15], Hodgkin’s lymphoma [16–18], B-cell malignancies [19, 20], brain tumours [21–25], prolactinoma [26, 27], melanoma [28–31], lung cancer [32, 33], colorectal cancer [34–36], gastric cancer [37] and liver cancer [38, 39].

Nevertheless, receptor expression pattern is not enough to predict the effect of estrogen or progesterone on a given tumour type. In general, ERα can induce estrogen-dependent proliferation while ERβ can inhibit it, but the overall biologic effect of estrogenic compounds on different cells, tissues and tumour types is determined by the complex interplay of ER subtypes, isoforms, ER-related receptors and heterodimerization, presence of transcriptional coactivators and corepressors, as well as estrogen and non-estrogen mediated phosphorilation of all the above-listed ’players’. As mentioned in the previous section, estrogen effect on the stroma, immune response and participants of tumour metastatization also modifies the hormonal effect in vivo. As the effect is therefore practically impredictable, experience and its proper interpretation in the preclinical and clinical setting is inevitable.

## Determinants of Progesterone Effect on Tissues

**Fig. 2:**
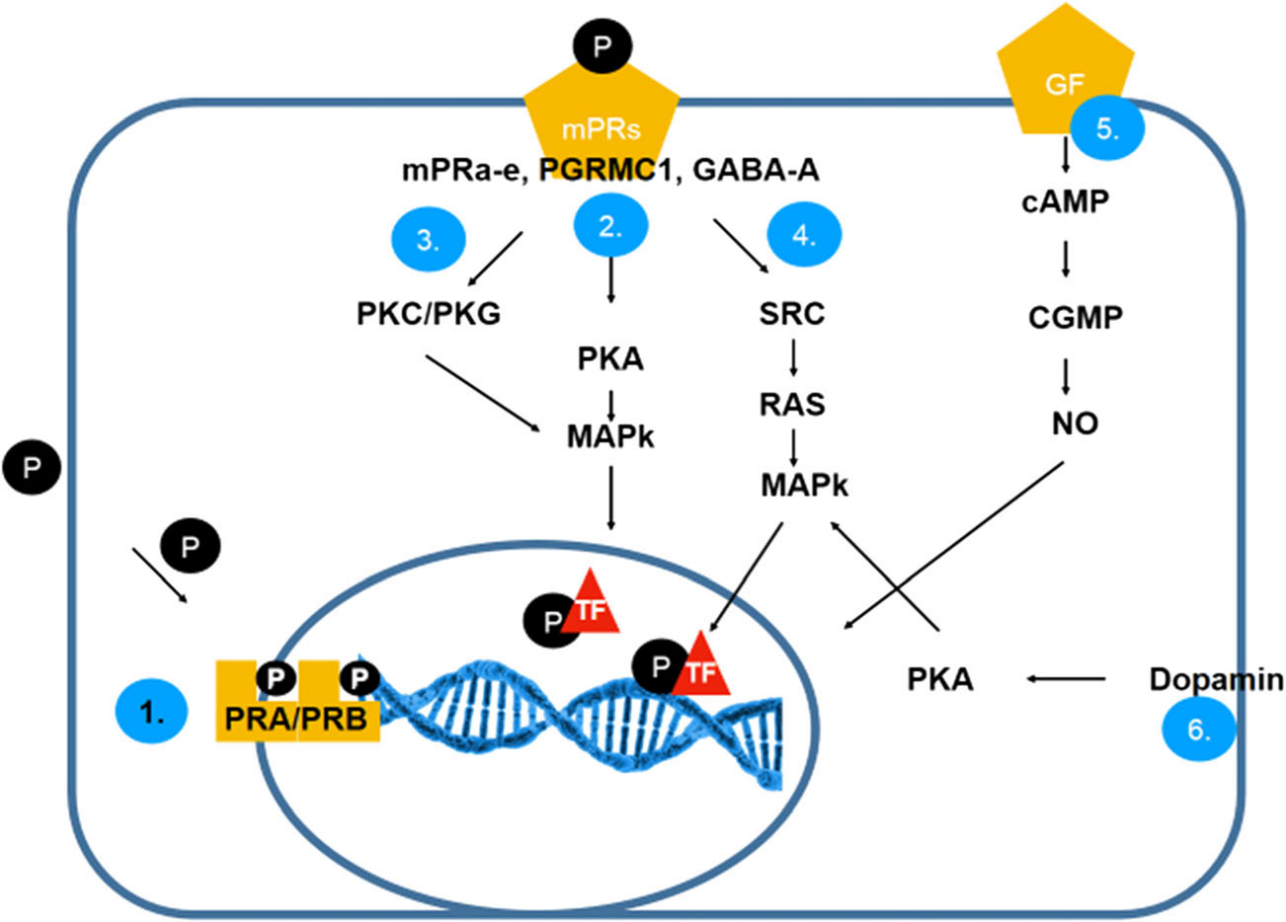
**Modes of action of progesterone receptors**. (1) Classical progesterone receptor activation (slower) through the classical nuclear progesterone receptors (PR-A/PR-B). (Progesterone + nuclear PRA/PRB, dimerization, direct DNA binding. ) Non-classical pathways are more complex. Effect of progesterone via cell surface membrane bound mPRs (PGRMC1,MPRab,GABAa), Ca^2+^ signalling and PKA/MAPK (2) activation or PKG/PKC/MAPK (3) activation. (4) Activation of SRC (tyrosine kinase) and MAPK cascade. (5) Growth factors (GnRH, neuropeptides and PGE2) can be effective through cAMP, cGMP, NO via nuclear PRs. (6) Dopamin mediated effects via PKA activation [40–42]

As both the symptoms and the long-term negative effects of menstrual cycle cessation are attributed mainly to estrogen deficiency, HRT needs to substitute estrogen. Yet, to prevent the oncologic risk of unopposed estrogen stimulation of the endometrium, progestagenic compounds are also used in patients with an intact uterus. As the progestagens used do not have a pure progesterone-like effect (except for micronised progesterone) but can also act as activators or inhibitors of different potency on other steroid hormone receptors (androgen, mineralocorticoid and glucocorticoid), prediction of E+P HRT effect on tissues simply based on receptor expression and signalling is nearly impossible. Progesterone signalling is not less complicated than estrogen signalling, and beyond that, estrogen and progesterone interaction, progesterone effect on the tumour stroma, immune cells and the events of metatstatization and the effect on other steroid hormone receptors combine to finally determine the overall impact that progesterone-containing HRT exerts on a certain malignant disease. Detailed discussion of progesterone signalling exceeds the scope of this review. Nuclear progesterone receptor (PR) A and B activation and progesterone response element (PRE) binding leads to ’classical’ progesterone signalling, but rapid progesterone effects are also mediated by membrane-bound PRs, cytoplasmic PRs and receptor-independent signalling via various signal transduction pathways, called ’non-classical’ progesterone signalling, as reviewed in detail by Garg and colleagues [40] and summarized in Fig. 2 [40–42].

Similarly to what was discussed above in detail with regard to estrogen, progesteron receptor expression pattern is also not enough to exactly predict the effect of progesterone on a given tissue or tumour type. Therefore, each cancer type and its relation to MHT requires an independent search of the literature. Thus, to make the most advantage of this work, we included in our search the most common tumour types, whose survivors are therefore the most probable to show up with the request of HRT. Childhood and young adult (ages 20 to 39) cancer survivors will present with premature ovarian insufficiency, whereas survivors of the most common female cancer types may come for a professional opinion regarding HRT at or after the natural age of menopause. The most common cancer types are listed in Table 1, based on the 2015-2016 data of the American Cancer Society.

**Table 1:** The most common cancer types in childhood, young adults and females. American Cancer Society, 2015-2016 [43]

Childhood cancer	Young adult cancer	Female cancer (new cases)
Leukemia	Breast cancer	Breast cancer
Brain and spinal cord tumours	Lymphoma (Hodgkin / non-Hodgkin)	Lung cancer
Neuroblastoma	Melanoma	Colorectal cancer
Wilms tumour	Sarcoma	Uterine corpus
Lymphoma (Hodgkin / non-Hodgkin)	Female genital tract cancers	Thyroid cancer
Rhabdomyosarcoma	Thyroid cancer	Non-Hodgkin lymphoma
Retinoblastoma	Testicular cancer	Melanoma
Bone cancer (osteosarcoma, Ewing sarcoma)	Colorectal cancer	Leukemia
	Leukemia	Pancreatic cancer
	Brain and spinal cord tumours	Kidney cancer

In this review, we are going to discuss the relation of MHT and various cancer types according to the following grouping:

1. Breast cancer
2. Gynecologic cancers
3. Other common non-gynecologic cancers

## Breast Cancer

One of the most commonly encountered clinical situation in the field of HRT in oncologic patients is the request of breast cancer survivors to relieve their menopausal symptoms. Their menopause can be the result of either chemo- or radiotherapy, or some type of antiestrogenic endocrine therapy. The general attitude is straightforward, as also stated in the guideline of the International Menopause Society: no hormone replacement therapy should be given to these patients [1]. Rather, non-hormonal methods, including lifestyle changes, behavioral therapy, gabapentine, venlafaxine or fluoxetine should be preferred [44]. Although the observational and case control studies before 2002 indicated no increased risk of recurrence [45–48], HABITS (Hormone Replacement Therapy After Breast Cancer – Is it Safe?), the first large randomized, controlled trial (RCT) of the field changed the attitude and has dominated professional approach ever since. HABITS was stopped in 2003 after 2.1 years as the results showed an increased risk of breast cancer recurrence (n=434, recurrence 26 cases in the HRT group vs. 7 cases in the non-HRT group, HR: 3.3) [49]. The other RCT at the time, the Stockholm Trial was also stopped based on the results of the HABITS trial, although its results showed no increased risk of recurrence, with a RR of 0.82 when the trial was prematurely stopped (n=379). However, there were significant differences between the two trials effecting the lymph node positivity and tamoxifen application. Although the original RCT results seemed to be conflicting, and even now no clear-cut conclusion can be drawn, trials of the last decade, including extended follow-up studies of HABITS and the Stockholm Trial have seemed to indicate an increased recurrance risk of breast cancer after different HRT regimens, with the relative risk (RR) of recurrence varying between 2.0 to 3.6 [50, 51]. In the extended follow-up of HABITS, Holmberg and colleagues found a RR of recurrence of 2.4 (n=442, mean HRT duration 24 months, follow-up 5 years, recurrence 22.2% HRT user vs. 8.0% non-user) [52]. The 10-year follow-up results of the Stockholm Trial also indicate an increased breast cancer recurrence risk. In the study of Fahlen and coworkers, a hazard ratio (HR) of 3.6 was detected for recurrence of the disease (n=378, mean HRT duration 26 months, recurrence HRT users 7,4% vs. non-users 2.1%) [53].

Not only estrogen + progestagen oral HRT regimens have been tested, but also tibolone, a compound that is metabolized to an estrogenic, progestagenic and androgenic isomer and is routinely used for MHT. The LIFT (Long-Term Intervention on Fractures with Tibolone) study proved that not only does tibolone decrease fracture risk in an osteoporotic postmenopausal population, but it also reduces invasive breast cancer risk significantly (odds ratio 0.32) [54]. The LIBERATE (Livial Intervention Following Breast Cancer: Efficacy, Recurrence and Tolerability Endpoints) Trial was conducted to assess the use of tibolone in breast cancer survivors. Although bone mineral density (BMD) and climacteric symptoms significantly improved, the trial was prematurely terminated because of the increased recurrence risk of breast cancer (n=3098, follow-up 3.1 years, breast cancer recurrence 15.2% with tibolone vs. 10.7% with placebo, HR: 1.4) [55–57]. Interestingly, cancer recurrence was only observed in the normal BMD group, suggesting a local estrogen effect unrelated to the circulating estrogen in the plasma. Unfortunately, due to the disapointing outcome of these prematurely halted trials, we could not get a clear vision on the safety of HRT in breast cancer survivors in the following decade.

Although HRT is generally contraindicated in breast cancer survivors, mainly based on the studies mentioned above, some points are worth consideration during decision-making.

1. Different molecules used for HRT and different regimens resulted in conflicting outcomes regarding cancer recurrence risk.
2. The effect of tibolone can be modified by the presence of ER and parallel endocrine oncotherapy: whereas recurrence risk was high when aromatase inhibitor (HR: 2.42) or GnRH analogue (HR: 2.29) was also used, no significant relative risk elevation was seen in ER negative (HR: 1.15) and SERM (tamoxifen) -treated (HR: 1.25) cases. Differences can be explained by the different antiestrogenic effects, resulting in up- or downregulation or blockage of ERs.
3. To relieve the symptoms of urogenital atrophy, local estrogen therapy is also commonly used. When used vaginally, a low-dose estrogen tablet delivers an annual amount of 1,14 mg estrogen, compared to the 182.5 mg delivered by the standard dose oral estrogen tablet [58]. Comparing vaginal estrogen user breast cancer survivors and non HRT user breast cancer survivors, Durna and colleagues found 9.1% recurrence in vaginal estrogen users versus 29.5% in non-HRT users (RR: 0.18; 0.04-0.75) [59]. Fahlen and colleagues compared the use of oral and local estrogen HRT in breast cancer survivors. Recurrence was detected in 7.4 % vs. 2.1 % of the two groups, respectively [60]. Mortality between the two groups, however, was not significantly different. It is generally accepted, that any form of local estrogen application is contraindicated during adjuvant aromatase inhibitor therapy, since the serum estrogen level has to be kept strictly at zero.
4. The duration of HRT seems also to be of significance, although direct conclusions can not be drawn here either. Studies covering longer periods of MHT after breast cancer (24-42 months) tend to report more recurrences and increased mortality [52, 60, 61], as compared to shorter periods of HRT (12-22 months) [59, 62].
5. A special situation arises in BRCA mutation positive women, following prophilactic bilateral salpingo-oophorectomy and thus becoming menopausal. In such situations, the clinician can face the following possibilities and can give the following suggestions according to Finch and colleagues [63]:
  If breasts are intact and there is no history of breast cancer: in both BRCA1 and BRCA2 mutants, MHT *can be offered* to the age of natural menopause (50 years of age).
  If prophylactic mastectomy has been performed but there is no history of breast cancer, MHT *should be offered* to the age of natural menopause.
  If the patient had breast cancer, MHT is *contraindicated*.
  In a recent international cohort study of BRCA-1 mutation women after prophylactic oophorectomy and with intact breast, the risk of breast cancer was found elevated when estrogen-progestin HRT was applicated, but the risk did not increase with estrogen-only therapy. This finding is in accordance with the observations with MHT and breast cancer risk in the natural menopause, and raise the indication of removing the uterus also at prophylactic oophorectomy in BRCA mutation women. [64]

## Gynecologic Cancers

### Ovarian Cancer

Although ovarian tumours can be histologically very various and thus general guidelines are almost impossible to make as far as MHT is concerned, the situation is a little more simple in clinical practice, since approximately 90% of ovarian malignancies are epithelial ovarian tumours. Several studies and their meta-analyses have shown that MHT either does not increase recurrence of the malignant disease or (in some studies, e.g. Mascharenas et al.) it even increases the ovarall survival of patients significantly. [65–72]

The follow-up periods of the studies cited vary between 42 months to 19 years, thus these results are true in the long run.

The use of MHT in more rare types of ovarian tumours have less strong evidence, given the smaller number of cases and thus the difficulty of collecting population based data. Usually it is stated that HRT after the treatment of germ cell tumours probably carries no additional recurrence risk [73]. Endometrioid ovarian cancer is usually mentioned as a type that might be sensitive to estrogen a thus avoidance of HRT is often suggested. [73, 74]

This, however, might seem to be illogical, considering the fact that endometrial adenocarcinoma survivors are candidates for HRT, as shown in the following section.

Another type of ovarian cancer where the general suggestion is to avoid HRT is granulosa cell tumour, the most common sex-chord stromal ovarian tumour. Although there is no direct evidence to prove or disprove the long-term negative effect of HRT on granulosa cell tumour survivors, considering the hormonally – endocrinologically active character of these tumours, it may be safer not to initiate HRT in these patients. [73, 75, 76]

### Endometrial Cancer

Endometrial cancer is estrogen sensitive in 90% of the cases (type I) and estrogen independent in only 10% (type II, most commonly serous papillary carcinoma). Although one could expect an increased recurrence rate after the initiation of hormone replacement therapy, the studies published over the past decades do not support this hypothesis. Several small observational studies have found consistently that recurrence rate and disease free survival were not worse, furthermore, in most cases they were found to be even better in HRT groups than non-HRT groups [77–81]. The studies were far not uniform: in most cases, stage I and II (in one study, III as well) patients were included. Follow-up times were 42-87 months, and HRT was either estrogen only or combined estrogen + progestagen. Similar results were found by both the only prospective randomized controlled trial of the field involving over 1200 patients [82] and a meta-analysis of nearly 900 HRT patients vs. 1100 controls [83]. The latter meta-analysis also shoved that estrogen+progestin HRT had a protective effect against cancer recurrence (OR: 0.23; 95% CI 0.08-0.66), whereas estrogen-only therapy did not show this effect (OR: 0.35; 95% CI 0.06-2.10).

As to when to start HRT, we can only refer to the studies listed above. HRT was initiated after between 1 to 60 months of disease free survival (after surgery), but in most cases the period that passed after surgery was between 3-12 months.

No specific studies can be found on HRT in survivors of estrogen-independent, agressive type II endometrial cancers, but as they are not sensitive to estrogen, it is logical to think that HRT use is not more dangerous in this disease than in the hormone sensitive histologic forms of endometrial cancer.

### Uterine Sarcoma

Uterine sarcomas include leiomyosarcomas, carcinosarcomas, adenosarcomas and endometrial stroma sarcomas. Endometrial stroma sarcomas overexpress estrogen and progesteron receptors and estrogen HRT and tamoxifen were reported to have an adverse effect on the disease outcome [84]. HRT in these tumours should therefore be avoided.

Although leiomyosarcomas very often overexpress estrogen and progesteron receptors [85], removal of the ovaries during hysterectomy did not improve the 5-year overall survival in the study published by Kapp and colleagues [86]. This may indicate, that leiomyosarcoma is not hormone sensitive and HRT may be given, as suggested by some authors [87]. Others, however, consider HRT too risky for these patients and vote against, given the lack of direct data to support or refute its safety [75].

In carcinosarcomas and adenosarcomas, HRT can be used [87].

### Cervical Cancer

80-90% of cervical cancer is squamous cell carcinoma, which is known not to be estrogen dependent. As Ploch demonstrated decades ago [88], HRT in either estrogen-only or a combined E+P form was advantageous for the patients. In the HRT group, recurrence was 20% and 5-year overall survival 80%, whereas in the control group these were found to be 32% and 65%, respectively.

10-20% of cervical cancer is cervical adenocarcinoma and its biological behaviour resembles endometrial cancer. This needs to be taken into consideration when making decision of HRT and probably it is beneficial to chose a combined E+P regime, as discussed above in the section about endometrial cancer. Undoubtedly, at least to our present knowledge, treated cervical cancer is not a contraindication for HRT.

### Vaginal and Vulvar Cancer

Most vaginal and vulvar cancers are squamous cell carcinomas, and behave similarly to squamous cell cervical cancer, as far as hormone insensitivity is concerned [89, 90]. Thus, MHT is not contraindicated.

Vaginal (clear-cell) adenocarcinoma most commonly developes after in utero diethylstilbestrol exposition, and vulvar adenocarcinoma can develop from Bartholin’s glands. These forms of adenocarcinoma are too rare and their relation to HRT is not well determined, therefore no clear guidance can be given. Some sporadic case reposts can be found in the literature implying a connection between unopposed estrogen exposition and vaginal adenocarcinoma [91], but reliable evidence is missing.

## Non-gynecologic Tumours

### Haematologic Malignancies

Malignant haematologic diseases include for example acute and chronic lymphoid and myeloid leukaemia, Hodgkin lymphoma, non-Hodgkin lymphoma and myeloma multiplex. The treatment of haematologic malignancies can cause premature ovarian insufficiency via both chemotherapy and stem cell transplantation. The beneficial effect of estrogen on haemopoietic cells has been showed in several ways. Lymphocyte precursors are regulated by sex steroids [92]; myeloid leukaemia cells express estrogen receptors [93] and their methylation is related to patient survival [94]; pregnancy is protective against the development of Hodgkin lymphoma[95]; former HRT decreases the risk of B-cell non-Hodgkin lymphomas in postmenopausal women[96]; estrogen influences the proliferation, differentiation and survival of B-linage precursors [97] and by decreasing local IL-6 production, improves the disease-free and overall survival in diffuse large-cell lymphomas [98, 99]. The studies that directly address the question of MHT after oncotherapy of malignant haematologic diseases found no increase in the recurrence of the disease or excess mortality, while significant alleviation of menopausal symptoms was demonstrated [100]. In summary, at least neutral effect of HRT on malignant haematologic diseases can be demonstrated, but most probably estrogen supplementation even exerts a positive effect on these diseases [101].

### Brain Tumours

Even in the past years, conflicting data can be found about the effect of HRT on the incidence of brain tumours in general and meningioma and glioma in particular. It, however, seems clear, that estrogen and progesterone can promote the growth of malignant brain tumours. It has been observed that meningiomas are more common in women, and grow faster in the luteal phase of the cycle and during pregnancy [102, 103], indicating the role of progesterone effect. 58-83% of meningiomas express progesterone receptor as opposed to 0-8 % expressing estrogen receptor [104, 105]. Inconsistent results of studies indicate various relationship between the different forms of HRT and brain tumours: MHT increases the risk of meningioma by 30-80 %, but not that of glioma [106]; meningiomas can grow as a result of progesterone, estrogen and androgen stimulus [107]; estrogen-only HRT, but not E+P HRT increased the risk of brain tumours, glioma and meningioma in a large UK database of women aged 50-79 [108]; HRT but not oral contraceptive use was associated with an increased meningioma risk [109]; progesterone-only contraception is associated with a shorter progression-free survival in premenopausal women with WHO Grade I meningioma [110]. Taken all these together, it seems to be clear that brain tumours, and especially meningioma and glioma, may be sensitive to estrogen and even more to progesterone, and hormones can stimulate their growth and recurrence, therefore HRT should be avoided in these patients.

#### Prolactinoma

Due to its well-established estrogen sensitivity, and the frequency of the clinical situation of requirement of HRT in prolactinoma patients, prolactin producing pituitary adenomas are discussed in a separate section. Traditionally, prolactinomas were considered a contraindication for estrogenic treatment including combined contraceptive pills and hormone replacement therapy. It is known, that clinically significant tumour growth is very rare (1-2%) during pregnancy and during low-dose (<30 ug) ethynil estradiol containing contraceptives in case of a microprolactinoma, whereas macroadenomas grow much more frequently in an estrogenic milieu, e.g. their 30-40% grow during pregnancy. The presence or history of a microprolactinoma should therefore not be considered a contraindication for HRT in the case of hypogonadism or in the presence of menopausal symptoms [111]. In case of a macroprolactinoma, thorough case-by-case evaluation is required, and if estrogen therapy is prescribed, and very close monitoring of the prolactinoma size and activity is necessary, also keeping in mind that the sequential form of HRT (or cyclic oral contraceptive use) masks the important symptom of ovulatory dysfunction and menstrual irregularities [111]. In contrast, it has not been proven that even higher doses of estrogen (a cumulative 19 mg/kg body weight) used in normoprolactinaemic patients for prolonged periods (5-15 years) would cause hyperprolactinaemia [112].

### Malignant Melanoma

Malignant melanoma is one of the most aggressive tumours. Its different biological behaviour in males and females is well known: men tend to have more rapid progression and earlier metastases, leading to increased mortality and decreased survival. The response of melanoma cells to female hormones, however, has been reported controversial. Some studies report protective effects of estrogen [113, 114], others found estrogen-only HRT increasing the risk of skin malignant melanoma, which was not the case for E+P combined HRT [115]. The contradiction can be solved if we consider the significance of estrogen receptor-β (ERβ) in malignant melanoma. While ERα is associated with a proliferative and tumour promoting effect, ERβ has an antitumour effect, through the inhibition of the PI3K/Akt pathway [116]. ERβ expression leads to a better prognosis, while decreased expression of the receptor results in poorer prognosis and the metastatic state of the disease [117–120]. This knowledge may lead to the use of ERβ expression determination of the tumour and the use of estrogen or ERβ agonists in the treatment of melanoma. Considering MHT, local, and thus probably ERβ-rich tumours should be handled differently from advanced, metastatic cases. In the former group, estrogen supplementation might even be advantageous, but at least will not have adverse effects, as demonstrated by a study of 206 patients suffering from stage 1 or 2 cutaneous melanoma and followed up for 5 years after surgery [121]. One out of the 83 HRT-receivers died of melanoma, whereas 22 of the 123 patients in the non-HRT group died of the disease by the end of the 5 years. The safety of MHT in advanced, metastatic melanoma malignum is unclear and no recommendation can be made regarding its use.

### Lung Cancer

Lung cancer in the past decades has also become one of the tumours considerded to be estrogen-dependent. Although ERβ generally starts anti-tumour signalling, in non-small cell lung cancer it has been established as a tumour promoter, most probably via interactions with receptor splice variants, EGFR receptor signalling and G-protein coupled estrogen receptors [122]. Several in vitro and in vivo studies have demonstrated the tumour promoting effect of estrogen in lung cancer, and the prognostic value of ERα and ERβ expression [122, 123].

Clinical studies have come to conflicting conclusions, although different settings make comparison difficult. Nevertheless, neutral [124], positive [125] and negative [126] correlation between lung cancer incidence and mortality have also been reported. Given that the direction of research is now the application of antiestrogenic agents and aromatase inhibitors in the treatment of lung cancer [127], it is logical that estrogen as part of MHT should not be used in lung cancer patients.

### Colorectal Cancer

Colorectal cancer presents in less severe forms in women than in men, indicating a protective effect of female hormones [128–130]. Indeed, studies of the field unanimously indicate that estrogen decreases the incidence of colorectal cancer and decreases the progression of the disease [131–136]. This is due to the presence of the ERβ exerting anti-tumour effect [137]. The latest research indicate that ERβ expression can even be used as a positive prognostic marker in the treatment of colorectal cancer [138–140]. In the light of these results, there is no doubt that MHT has a positive effect on colorectal cancer and survivors of the disease suffering from the symptoms of ovarian insufficiency should be offered MHT.

### Kidney Cancer

Several studies have concluded that no relation between MHT and kidney cancer can be established [141–145], therefore survivors of kidney cancer can be offered HRT if indicated.

### Gastric Cancer

ER positivity has long been known to be associated with poor outcome of gastric cancer treatment: ER+ cases are generally more disseminated and less differentiated [146]. Postperative survival rates are significantly worse in ER+ vs. ER- cases (15 % vs. 62%) as well as in progesterone receptor positive cases [147]. In the past years, ERα and ERβ isoforms have been identified as prognostic markers of gastric cancer [148]. For example, ERα66 has been reported in poorly differentiated gastric cancer, ERα36 is found more often in lymph node metastases, and ERβ1 is associated with low grade tumours [148]. Besides ERα, androgen receptor expression was also found to be associated with a poor prognosis and decreased progress free survival [149]. Beyond the hormone receptors and in the lack of clinical trials, two further – and contradicting – clinical research papers are worth consideration. Kim and colleagues reported that the clinical outcome and the overall survival was much poorer among female gasctric cancer patients than among males [150]. Brusselaers and colleagues, however, found a decreased esophageal and gastric adenocarcinoma incidence among ever-users of estrogen-only and E+P combined HRT, too, as compared to non-users of HRT, in a Swedish population of approximately 1.150.000 [151]. Taking all these data into consideration, starting MHT to gastric cancer survivors should rather be avoided, and especially so in case of estrogen or progesterone receptor positive tumours.

### Liver Cancer

It has been almost 30 years since Adami and colleagues reported a decreased risk of hepatocellular cancer following MHT [152]. The role of estrogen can be a strong antioxidant and anti-inflammatory effect, thus preventing fibrosis as a key step towards liver carcinogenesis [153]. Estrogen can also inhibit the progression of hepatitis B virus infection, thus inhibiting hepatocellular carcinoma [154]. These effects add up to a decreased incidence of hepatocellular cancer and better overall survival among patients that received MHT [155].

### Bladder Cancer

Bladder cancer has been known to be more aggressive in women then in males. 12-18% of bladder cancers are ER positive, and ER+ tumours tend to be of higher grade than ER- ones [156]. Former use of MHT was reported to double the risk of bladder cancer [157]. Both in vitro and in vivo studies indicate the role of estrogen in the initiation and progression of bladder cancer [158]. Applying the selective estrogen modulator tamoxifen in bladder cancer seems to be effective, although only initial clinical pilot studies are available [159, 160]. Combination of antiestrogen + BCG therapies are also experimented with [161]. In summary, bladder cancer should be considered an estrogen sensitive tumour, and although no direct clinical evidence is available, MHT should not be prescribed to survivors of this type of malignancy.

### Thyroid Cancer

Thyroid malignancy is more common in women than in men. Several epidemiologic studies have been carried out, but no strong evidence of relation between MHT and differentiated thyroid cancer could be found [162–168]. This is not changed even by the fact that in the past years, both positive and negative associations have been reported between the expression of estrogen and progesteron receptors and the outcome of thyroid cancer [169, 170]. Thyroid cancer survivors often receive suppressive doses of thyroxin substitution to maintain subclinical hyperthyroidism in order to suppress TSH and thus decrease recurrence risk. Is is known that subclinical hyperthyroidism increases the risk of cardiovascular disease and accelerated bone loss, therefore estrogen substitution, that counteracts both of these negative effects in the case of menopause is even more indicated. Increased frequency of breast cancer among thyroid cancer survivors has been reported but mostly it is attributed to the late effect of radioiodine therapy of the primary malignancy of the thyroid, although no definitive consesus has been reached yet [171]. In summary, former thyroid cancer should not be considered a contraindication to MHT.

### Pancreatic Cancer

Based on 27 case-control and cohort studies, Tang and colleagues made a meta-analysis which revealed that the risk of pancreatic cancer is not associated with exogenous hormone use (oral contraceptives or MHT) or menstrual factors (age at menarche, age at menopause, hysterectomy or oophorectomy) [172]. Pancreatic cancer treatment in the patient’s history does not contraindicate MHT.

## Conclusions

As a result of rapid advance in oncology, both the diagnosis and the treatment of malignant tumours has been constantly improving, leading to the increasing survival of oncologic patients. More and more of them live long enough to reach either the natural age of menopause or, as a side effect of their oncotherapy, experience the cessation of their gonadal function, leading to premature ovarian insufficiency. Thus, an ever increasing number of cancer survivors search endocrinologic help in the form of the replacement of their missing female hormones. Often it is not the endocrinologist who is first asked by the patient, but other members of the professional staff involved in her oncologic care, including the oncologist, gynecologist, general practitioner, or even the radiologist or the oncologic nurse. The general attitude has been defensive: ignorance of the latest results in the field of menopausal hormone therapy and false concepts dating back to the WHI (Women’s Health Initiative) Study results almost two decades ago [173]. The misinterpretation of the results of this study are well known among the specialists of the field of menopausal medicine and will not be discussed here. The net outcome, however, became an irrational fear of female hormone replacement, both by the general population and medical professionals. It might has seemed the logical and safe conclusion to many physicians that it is better to avoid HRT, because this attitude definitely causes no harm, whereas the decision of prescribing estrogen with or without progestins for a patient might bear oncologic and cardiovascular risks, may lead to litigation in case of a potentially related complication, and requires meticulous and continuous self-education in the field not to miss the latest evidence. The major mistake in this reasoning is the baseline theory of not harming by avoiding HRT. It was known even before the WHI results that premature menopause and hypogonadism decreases the life expectancy of women by years through its skeletal and cardiovascular effects, and this negative effect correlates with the length of the hypoestrogenaemic period. 17 years of untreated hypogonadism add up to losing 2 life-years [4], and every year of delay of menopause decreases cardiovascular mortality risk by 2% [174]. The quality of a life lived with severe menopausal symptoms is yet another matter not to forget. Therefore, *it is not prescribing HRT for a young patient suffering from premature ovarian insufficiency (POI) that requires professional explanation* - as it is clearly stated also by the latest guidelines of the International Menopause Society [1] and the Global Consensus Statement on MHT [175]. *It is the denial of HRT*, a decision proved to harm the patients health and decrease her life expectancy, *that needs to be supported by evidence* and should be weighed againts the risks (oncologic or other) of HRT. Denying HRT ’just to be on the safe side’ is unacceptable – we can only make this maleficent decision if the definitive harm caused to our patient by it is smaller than the suspected harm of HRT.

Yet, it is not easy to assess the HRT-related extra risks of HRT in cancer survivors. Practically, every tumour is a new and potentially unprecedented entity, as far as its oncogenic mutations, hormone receptor status and biological beviour are concerned, even if medical science tries to group tumours according to organ, histology or molecular characteristics. The variability of the stage, grade and former oncotherapy received by the patient complicates decesion making even further. It is clear that deciding for or against HRT should always be individualized and decision should be made together with the patient. The risk-benefit assessment can be based on a wide range of scientific evidence of different quality, depending on their availability: from in vitro studies to clinical investigations, and the latter ranging from individual case reports to meta-analysis of several randomized prospective trials, and even indirect evidence (e.g. epidemiologic data of tumour behaviour in the different sexes, during hormonally altered periods like in pregnancy, or HRT effect on tumours not in cancer survivors etc.). In this review we have tried to collect data about the most common types of tumours, in an attempt to help clinical decisions (Table 2). Based on the evidence gathered so far, adequately chosen female hormone substitution can be clearly advantageous in some cancers (e.g. endometrial cancer, haematologic malignancies or colorectal carcinoma) and harmful in hormone dependent others (e.g. breast cancer, meningioma or ER+/PR+ gastric cancer). In certain tumours or in their subgroups, the risk of HRT seems to outweigh the potential benefits and thorough individualized decision-making is necessary (e.g. lung cancer or bladder cancer), whereas in many tumour types MHT is neutral for the malignant disease and thus it should not be denied (e.g. cervical cancer, kidney tumours or pancreatic cancer). In many situations, however, when more rare cancer types are faced, individual search of the literature for the specific tumour will be necessary. Clinical evidence of recurrence risk of the given cancer type, weighed against the negative long-term effects of avoiding HRT, the general state and life-expectancy of the patient, the severity of her hypoestrogenic and other symptoms, and her subjective fear of the recurrence of the disease, which is understandably always dominant in cancer survivors, will help to find the best solution.

**Table 2:** Categories of cancer types according to oncologic risk (recurrence, progression) of hormone replacement therapy

HRT:	Adavantageous	Neutral (no known negative effect)	Negative effect in certain setting (relative contraindication)	Disadvantageous (contraindicated)
Breast cancer		BRCA 1/2 mutation without breast cancer		Breast cancer
Gynecologic cancers	Endometrial cancer (E2 dependent, type I) – E+P advantageous, E-only: neutral	• Endometrial cancer (E2 independent, type II) • Uterinal carcinosarcoma • Uterinal adenosarcoma	Leiomyosarcoma	Endometrial stroma sarcoma
		Ovarian cancer (epithelial, germ cell tumour)	Some ovarian tumours: • endometrioid ovarian cancer (?) • granulosa cell tumour	
	Cervical adenocarcinoma (E+P)	Cervical cancer (squamous cell)		
		Vaginal cancer (squamous cell) (adenocarcinoma??)		
		Vulvar cancer (squamous cell) (adenocarcinoma??)		
Non-gynecologic cancers	Haematologic malignancies (leukaemias, lymphomas)	• Microprolactinoma • Macroprolactinoma (?? – close follow-up required if on HRT)	Brain tumours	• Meningioma • Glioma
	Malignant melanoma (local, cutaneous)		Malignant melanoma (advanced, metastatic)	
	Colorectal cancer		Lung cancer	
	Liver (hepatocellular) cancer	Kidney cancer	Gastric cancer	Gastric cancer (ER+, PR+)
		Thyroid cancer	Bladder cancer	Bladder cancer (ER+)
		Pancreatic cancer		
